# Integrative taxonomy of the genus *Longidorus* (Nematoda: Longidoridae) reveals two new species in the Mediterranean Basin

**DOI:** 10.1186/s40851-025-00254-3

**Published:** 2025-11-07

**Authors:** Rosana Salazar-García, Juan E. Palomares-Rius, Aadil Bajoub, El Amine Ajal, Idriss Ouled-Bouallala, Carolina Cantalapiedra-Navarrete, Pablo Castillo, Antonio Archidona-Yuste

**Affiliations:** 1 Institute for Sustainable Agriculture (IAS), Department of Crop Protection, Spanish National Research Council (CSIC), Avenida Menéndez Pidal s/n, Campus de Excelencia Internacional Agroalimentario, Córdoba, 14004, ceiA3 Spain; 2 Forestal y del Desarrollo Rural Sostenible, Programa de Doctorado de Ingeniería Agraria, Universidad de Córdoba, Alimentaria, Spain; 3 Laboratory of Food and Food By-Products Chemistry and Processing Technology, National School of Agriculture in Meknès, km 10, Haj Kaddour Road, B.P. S/40, Meknès, Morocco; 4 UPR of Pharmacognosy, Faculty of Medicine and Pharmacy of Rabat, Mohammed V University, BP 6203, Rabat, 1000 Morocco

**Keywords:** COI MtDNA, D2-D3 of 28S rRNA, Taxonomic study, ITS1 rRNA, Morphology, Olive

## Abstract

The needle nematode genus *Longidorus* comprises approximately 194 species of polyphagous plant ectoparasites distributed worldwide, some of which serve as vectors for plant viruses. However, the high species diversity and conserved morphology of these nematodes pose significant challenges for accurate identification of species. To address this issue, we conducted an integrative taxonomic study across 264 sites in major olive-growing regions (Greece, Morocco, Italy, Portugal, and Spain) of the Mediterranean Basin, including nearby patches of natural vegetation. Herein, we describe two new species, *Longidorus olearum* sp. nov. and *Longidorus morocciensis* sp. nov., and report *Longidorus oakgracilis* in Portugal for the first time. We performed a comprehensive study that integrates morphological and morphometric traits with molecular data from nuclear ribosomal RNA (rRNA) genes (D2-D3 expansion segments of 28 S, ITS1, and partial 18 S) and a mitochondrial DNA (mtDNA) marker, specifically the Cytochrome c oxidase subunit I (COI). The results of our phylogenetic analyses provided robust support for the delimitation of the newly described species, *L. olearum* sp. nov. and *L. morocciensis* sp. nov., and further clarified of three previously recognized species within the genus: *L. magnus*, *L. oakgracilis*, and *L. vineacola*. Phylogenetic relationships inferred from ribosomal and mitochondrial markers revealed that the majority of *Longidorus* species from the Mediterranean Basin clustered within subclades of Clade I. The phylogenetic placement of these species demonstrated strong congruence across lineages, corroborating previous studies on the genus. These findings contribute to a broader understanding of *Longidorus* biodiversity in the Mediterranean region and highlight the need for further intensive and wide-ranging nematological surveys.

## Introduction

The needle nematode genus *Longidorus* comprises long to very long nematodes (2–12 mm) with elongated, needle-like odontostyle ranging from 42 μm in *L. brevis* from Senegal Swart et al. 1996 [1] to 180 μm in *L. tarjani* Siddiqi, 1962 [2] (Florida, USA). It currently includes approximately 194 species of polyphagous, plant-parasitic nematodes distributed worldwide [3–11]. To date, only about 6% of these species have been identified as plant virus vectors (specifically, carrying nepoviruses) [12], including *L. apulus* Lamberti and Bleve-Zacheo, 1977 [13], *L. arthensis* Brown et al. 1994 [14], *L. attenuatus* Hooper, 1961 [15], *L. caespiticola* Hooper, 1961 [15], *L. diadecturus* Eveleigh and Allen, 1982 [16], *L. elongatus* (de Man, 1876) [17] Thorne and Swanger, 1936 [18], *L. fasciatus* Roca and Lamberti, 1981 [19], *L. leptocephalus* Hooper, 1961 [15], *L. macrosoma* Hooper, 1961 [15], *L. martini* Merny, 1966 [20], and *L. profundorum* Hooper, 1965 [21].

The genus’ high species richness, coupled with generally conserved morphology and morphometrics, poses significant challenges for accurate identification, impeding both phytopathological and ecological research. Moreover, phenotypic plasticity within *Longidorus* results in pronounced intraspecific morphometric variability and subtle diagnostic distinctions. As a result, integrative taxonomy has become essential for reliable delimitation of species. Over the last decade, this approach has proven effective in uncovering cryptic diversity within the genus [3, 4].

Due to the limited morphological variability and frequent detection of only juvenile stages in soil samples, DNA barcoding techniques have increasingly relied on nuclear ribosomal and mitochondrial markers. Advances in molecular taxonomy now offer powerful tools for *Longidorus* identification [3–11]. Integrating multiple markers-particularly the D2–D3 expansion segments of 28 S rRNA, ITS1 rRNA, and the cytochrome oxidase subunit I (COI) gene-with morphometric and morphological species delimitation has proven highly effective in resolving species complexes within the genus [4]. These markers have also been instrumental in elucidating the phylogenetic relationships among *Longidorus* species over the past decade [3, 10, 11, 22–24].

Olive (*Olea europaea* (L.) subsp. *europaea* (L.)) is a major crop in the Mediterranean countries and was one of the first fruit trees cultivated in the Mediterranean Basin [25]. Olive is a crop adapted to the Mediterranean agroclimatic conditions and can be distinguished from their wild relatives by larger fruits and higher oil content of the fleshy oil-containing mesocarp. The edible oil and fruit products of cultivated olive trees are prized worldwide [25]. More than 80% of about 12 million hectares of olive grown worldwide were located in Mediterranean countries, including North Africa. The wide production of olive oil is concentrated in the Mediterranean area with Spain being the largest producer in the world [25].

Previous reports include three species in Greece, six in Italy, 10 unidentified populations in Morocco, two in Portugal, and 13 in Spain [22, 23, 26–28]. During the present study, we carried out a multinational survey across the Mediterranean Basin in spring 2023, and detected nine *Longidorus* populations in three of the five countries sampled: Morocco, Portugal and Spain.

The specific objectives of this study were: (i) to perform detailed morphological and morphometric characterization of the nine *Longidorus* populations collected from olive groves and nearby natural habitats in Morocco, Portugal, and Spain, and to compare them with other known species of the genus; (ii) to characterize these populations molecularly using D2–D3 segments of 28 S rRNA, ITS1 rRNA, partial 18 S rRNA, and COI gene sequences; and (iii) to infer the phylogenetic relationships of the identified species in the context of currently available *Longidorus* molecular data.

## Materials and methods

### Sampling and morphological characterization

Surveys were conducted during the olive flowering period in spring 2023, encompassing 264 sampling sites distributed across 44 olive groves and adjacent patches of natural vegetation. These sites were located within key olive-producing regions of the Mediterranean Basin, including Greece, Morocco, Italy, Portugal, and Spain. At each site, soil samples were collected from both cultivated olive groves and the nearest patch of natural vegetation, ensuring identical environmental conditions across paired samples.

Soil sample were obtained from the rhizosphere of host plants using a hoe, targeting the upper 50 cm of soil beneath four to five arbitrarily selected plants at each sampling site. From each bulk sample, a 500 cm³ subsample was processed for nematode extraction using centrifugal flotation [29]. Extracted specimens were heat-killed, fixed in a solution of 4% formaldehyde + 1% propionic acid, and processed into pure glycerin using Seinhorst’s method [30].

Light micrographs and measurements of the nine nematode populations were obtained using a Leica DM6 compound microscope equipped with a Leica DFC7000 T digital camera (Wetzlar, Germany). Morphological assessments included key diagnostic characteristics such as de Man indices, body length, odontostyle length, lip region width, tail length and shape, and guiding ring distance from the anterior end [31]. Specimens were mounted in glycerin for detailed examination.

Nematodes were identified at the species level using an integrative approach combining morphological analyses of females and J1–J4 stage juveniles [31] in combination with molecular analyses for efficient species delineation [7]. For the unidentified Longidorus population from Spain, a total of 37 nematodes were examined, including 18 adult females and 19 juveniles (first- to fourth-stage), mounted on microscope slides in glycerine. Additionally, 10 specimens were processed for DNA sequencing. For the unidentified *Longidorus* population from Morocco, a total of 35 nematodes were examined, including 15 adult females and 20 juveniles (first- to fourth-stage), mounted on microscope slides in glycerine. Eight of these specimens were additionally processed for DNA sequencing.

### DNA extraction, PCR, and sequencing

All *Longidorus* populations detected in this study were subjected to molecular characterization. In each case, DNA extraction was performed from individual specimens, ensuring that all molecular markers analyzed originated from the same single DNA-extracted specimen per PCR tube, minimizing the risk of sampling error. To prevent misidentification in cases where multiple *Longidorus* populations co-occurred within the same soil sample (Table 1), single nematodes were temporarily placed in a drop of 1 M NaCl containing glass beads, preventing physical damage, and studied closely.

**Table 1:** *Longidorus* spp. Recovered in the present study, their location and related plant, with the accession numbers assigned to each sequenced locus

Species	Sample code	Location	Host	D2D3	ITS	18 S	COI
*Longidorus olearum* sp. nov.	SO01	Sabiote, Jaén (Spain)	*Olea europaea* ssp. *europaea* (cultivated olive)	PV440517-PV440526	PV448530-PV448531	PV440575-PV440581	PV432580-PV432581
*Longidorus morocciensis* sp. nov.	ZN43	Meknès, region Fez-Meknès (Morocco)	*Olea europaea* ssp. *maroccana* (wild olive)	PV440527-PV440534	PV448532-PV448539	PV440582-PV440583	PV432582-PV432583
*Longidorus magnus*	SO14	Monturque, Córdoba (Spain)	*Olea europaea* ssp. *europaea* (cultivated olive)	PV440535	-^a^	-	-
*Longidorus magnus*	SO12	Carcabuey, Córdoba (Spain)	*Olea europaea* ssp. *europaea* (cultivated olive)	PV440536	-	-	-
*Longidorus magnus*	SO07	Villanueva del Arzobispo, Jaén (Spain)	*Quercus ilex* ssp. *rotundifolia* (oak)	PV440537	-	-	-
*Longidorus magnus*	SO09	Santisteban del Puerto, Jaén (Spain)	*Olea europaea* ssp. *europaea* (cultivated olive)	PV440538	-	-	-
*Longidorus oakgracilis*	SO20	Vila do Conde, Porto (Portugal)	*Olea europaea* ssp. *europaea* (cultivated olive)	PV440539	-	-	-
*Longidorus oakgracilis*	ARR2	Figueira de Castelo Rodrigo, Guarda (Portugal)	*Olea europaea* ssp. *europaea* (cultivated olive)	PV440540	-	-	-
*Longidorus vineacola*	SO05	Chiclana de Segura, Jaén (Spain)	*Retama sphaerocarpa* (globe-fruited retama)	PV440541	-	-	-

^a^ not sequenced

Nematode DNA extraction followed the protocol described by Subbotin et al. 2000 [32]. Briefly, individual nematodes were cut with a scalpel in a 20 µL drop of PCR buffer (ThermoPol^®^, Biolabs, New England, USA), to which 2 µL of proteinase K (600 µg/mL) was added. The tubes were frozen at−80 °C for 15 min, followed by consecutive incubations at 65 °C for 1 h and 95 °C for 10 min. Samples were then centrifuged at 16, 000 × g for 1 min and stored at−20 °C until use in PCR.

The D2-D3 expansion segments of the 28 S rRNA gene were amplified using primers D2A (5′-ACAAGTACCGTGAGGGAAAGTTG−3′) and D3B (5′-TCGGAAGGAACCAGCTACTA−3′) [33].The ITS1 region was amplified using the forward primer 18 S (5′-TTGATTACGTCCCTGCCCTTT−3′) and the reverse primer 26 S (5′-TTTCACTCGCCGTTACTAAGG−3′)[34]. The partial 18 S rRNA gene was amplified using primers 988 F (5′-CTCAAAGATTAAGCCATGC−3′), 1912R (5′-TTTACGGTCAGAACTAGGG−3′), 1813 F (5′-CTGCGTGAGAGGTGAAAT−3′), and 2646R (5′-GCTACCTTGTTACGACTTTT−3′) [35].The mitochondrial COI gene fragment was amplified using primers COIF (5′-GATTTTTTGGKCATCCWGARG−3′) and COIR (5′-CWACATAATAAGTATCATG−3′) following the protocol of Lazarova et al. 2006 [36].

All PCRs were performed under the conditions described by Archidona-Yuste et al. [28]. Amplified PCR products were subsequently purified using ExoSAP-IT (Affymetrix, USB Products) and sequenced on a 3130XL genetic analyzer (Applied Biosystems, Foster City, CA, USA) using the BigDye Terminator Sequencing Kit v.3.1 (Applied Biosystems). Sequencing was conducted at the StabVida facility (Costa da Caparica,Portugal).

Newly obtained sequences were submitted to the National Center for Biotechnology Information (NCBI) under accession numbers listed in Table 1 and associated phylogenetic trees. Sequence chromatograms for all four markers (D2-D3 expansion segments of 28 S, ITS1, 18 S rRNA, and COI mtDNA) were analyzed using DNASTAR LASERGENE SeqMan v.7.1.0. Species identity of the DNA sequences was confirmed using BLAST [37].

### Phylogenetic analyses

Phylogenetic reconstruction was performed using D2–D3 expansion segments of the 28 S rRNA, ITS1 rRNA, partial 18 S rRNA, and mitochondrial COI gene sequences from the nine recovered *Longidorus* populations, along with available *Longidorus* accessions from the National Center for Biotechnology Information (NCBI). Outgroup taxa were selected based on previously published studies [3,4,35,38,39] to ensure comprehensive molecular variation within the analyzed sequences [40].

Multiple sequence alignments were conducted using the FFT-NS-2 algorithm of MAFFT v.7.450 [41]. Alignments were visualized and manually edited in BioEdit v.7.2.5 [42], where poorly aligned positions were trimmed using a light filtering strategy (up to 20% of alignment positions). This approach minimizes the impact on tree accuracy while reducing computation time, as suggested by Tan et al. 2015 [43], since automated filtering methods often degrade single-gene phylogenetic inference. Phylogenetic analyses were performed using Bayesian inference (BI) in MrBayes v.3.1.2 [44]. The best-fit model of DNA evolution was determined using JModelTest v.2.1.7 [45] under the Akaike information criterion (AIC). The best-fit models, along with estimated base frequencies, the proportion of invariable sites, gamma distribution shape parameters, and substitution rates, were then implemented in MrBayes for phylogenetic analysis. The following evolutionary models were applied: General Time-Reversible model with invariable sites and gamma distribution (GTR + I + G) for the D2-D3 expansion segments of 28 S rRNA; symmetrical model with invariable sites and gamma correction (SYM + I + G) for the partial 18 S rRNA; and one-parameter model with invariable sites and gamma distribution (TPM2uf + I + G) for the partial COI gene. All Bayesian analyses were run separately for each dataset using four chains over 10 × 10⁶ generations, with Markov chain sampling at intervals of 100 generations. Two independent runs were conducted per dataset. After discarding the first 30% of samples as burn-in and assessing convergence, the remaining trees were retained for further analysis. A 50% majority-rule consensus tree was generated, with posterior probabilities (PP) assigned to each relevant clade. Phylogenetic trees were visualized using FigTree v.1.4.4 [46]. All alignments and original tree files generated during the phylogenetic analyses are publicly accessible at the Zenodo repository (10.5281/zenodo.15391936).

### Morphological characterization of Longidorus species

**Fig. 1:**
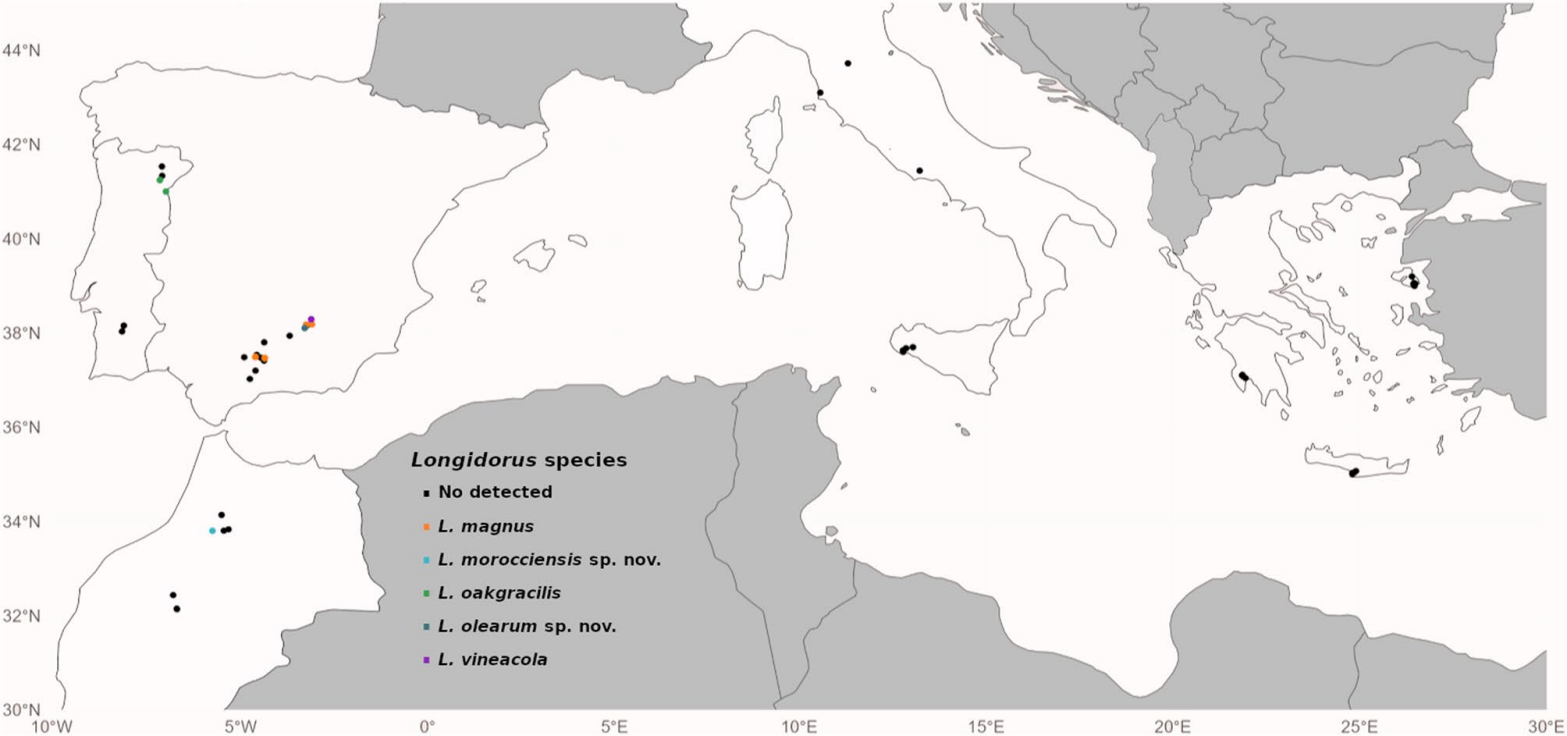
Geographic distribution of the sampling sites of olive groves in the Mediterranean Basin (black dots), and needle nematode species of the genus *Longidorus* detected in the present fieldwork

Nine of 264 soil samples (3.41% overall prevalence) collected from olive groves and adjacent patches of natural vegetation across five Mediterranean Basin countries tested positive for *Longidorus* spp. (Fig. 1; Table 1), with only one species detected per positive sample. Nematodes were recovered in Portugal, Morocco, and Spain, but were absent from samples collected in Italy and Greece. Most positive samples contained low densities of needle nematodes (1–4 individuals per 500 cm³ of soil), except for a notably high population of *L. vineacola* Sturhan & Weischer, 1954 [47] (200 individuals per 500 cm³ of soil) collected from globe-fruited retama (*Retama sphaerocarpa* L.) in Chiclana de Segura, Jaén, Spain. Detailed morphological, morphometric, and molecular analyses of these nine populations revealed five distinct species: three previously described (*L. magnus* Lamberti et al. 1982 [48], *L. oakgracilis* Cai et al. 2020b [49], and *L. vineacola*), and two new, undescribed species of the genus -*L. olearum* sp. nov. and *L. morocciensis* sp. nov., which are described herein.

### Molecular characterization of Longidorus species

Amplification of the D2–D3 expansion segments of the 28 S rRNA, ITS1 rRNA, partial 18 S rRNA, and the mitochondrial COI gene from both novel and previously described *Longidorus* species yielded single fragments of approximately 900 bp, 1100 bp, 1800 bp, and 400 bp, respectively, as estimated by gel electrophoresis. In total, 25 sequences of the 28 S D2–D3 expansion segments (PV440517–PV440541), 11 ITS1 rRNA sequences (PV448530-PV448539), nine 18 S rRNA sequences (PV440575–PV440583), and 13 COI sequences (PV432508-PV432520) were obtained (Table 1).

Low intraspecific sequence variation was observed for the D2-D3 region in *L. olearum* sp. nov. (PV440517-PV440526) and *L. morocciensis* sp. nov. (PV440527-PV440534), differing by 0–2 bp and 0–1 indels (99% similarity). The D2–D3 sequences of *L. olearum* sp. nov. and *L. morocciensis* sp. nov. shared 93.0–93.6% similarity, differing by 48–50 bp and 11–12 indels. Compared with other *Longidorus* species in GenBank, both new species showed 93% similarity to *L. iberis* (MW376567), *L. tabernensis* (MK941195), and *Longidorus* sp. 3 SAS-2014 (KF242335), differing by 53–56 nucleotides and 11–13 indels. Sequences from *L. magnus* (PV440535-PV440538), *L. oakgracilis* (PV440539-PV440540), and *L. vineacola* (PV440541) showed 99% identity to corresponding GenBank accessions (HM921361, MK941192, KT308873, respectively).

ITS1 rRNA was amplified from two specimens of *L. olearum* sp. nov. (PV448530–PV448531), which differed by 13 bp with no indels (98.8% similarity). For *L. morocciensis* sp. nov., eight sequences (PV448532-PV448539) exhibited very low intraspecific variation, differing by 0–6 bp with no indels (99.4–100% similarity). However, ITS1 sequences from both species showed limited similarity to other *Longidorus* ITS1 sequences in NCBI (coverage < 25–45%), and only moderate similarity to each other (78.7–79.3%), differing by 93 bp and 27 indels with coverage under 57%.

Seven partial 18 S rRNA sequences from *L. olearum* sp. nov. (PV440575–PV440581) showed minimal intraspecific variation (0–7 bp, 0 indels; 99% similarity), while no intraspecific variation was found in the two sequences of *L. morocciensis* sp. nov. (PV440582-PV440583). These sequences showed 98–99% similarity with most *Longidorus* species in NCBI. Comparisons between the new species showed 98.6–98.8% similarity, differing by 13–20 bp and one indel.

In the mitochondrial COI region, six sequences of *L. olearum* sp. nov. (PV432508-PV432513) differed by only 0–1 bp and 0–1 indels (99% similarity), while seven sequences of *L. morocciensis* sp. nov. (PV432514–PV432520) differed by 0–3 bp and 0–1 indels (99% similarity). *Longidorus olearum* sp. nov. COI sequences showed 78.1–78.7% similarity with *L. iliturgiensis* (MH454065), *L. maginicus* (OL471046), and *L. vineacola* (NC_033867), differing by 67–72 bp and up to 1 indel. Comparison with *L. morocciensis* sp. nov. revealed 78.3–79.0% similarity (differing by 56–61 bp, no indels). In contrast, *L. morocciensis* sp. nov. was most similar to *L. alvegus* (KY816661; 79.3%), *L. olearum* sp. nov. (78.3–79.0%), *L. iranicus* (KY816677; 78.1%), and *L. apulus* (KY816663; 74.4%), differing by 61–64 bp and up to six indels.

### Phylogenetic relationships among Longidorus species

**Fig. 2:**
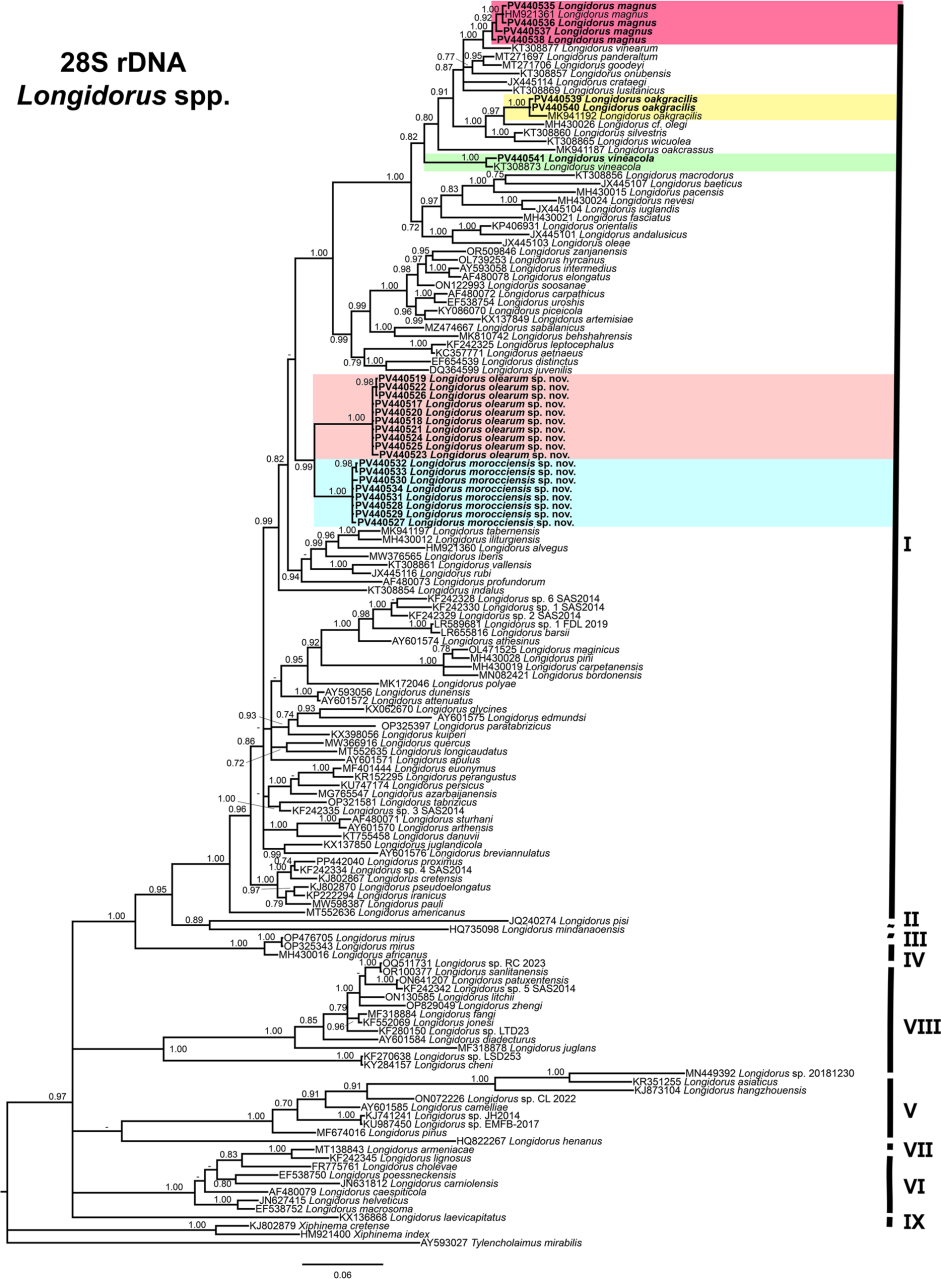
Phylogenetic relationships of *Longidorus* spp. from the Mediterranean Basin within the genus *Longidorus*. Bayesian 50% majority rule consensus tree as inferred from D2–D3 expansion segments of 28 S rRNA gene sequence alignment under the general time-reversible model with invariable sites and gamma distribution model (GTR + I + G). Posterior probabilities of more than 0.70 are given for appropriate clades. Newly obtained sequences in this are shown in bold. The scale bar indicates expected changes per site, and the colored boxes indicate the clade association within *Longidorus* species analyzed in this study

**Fig. 3:**
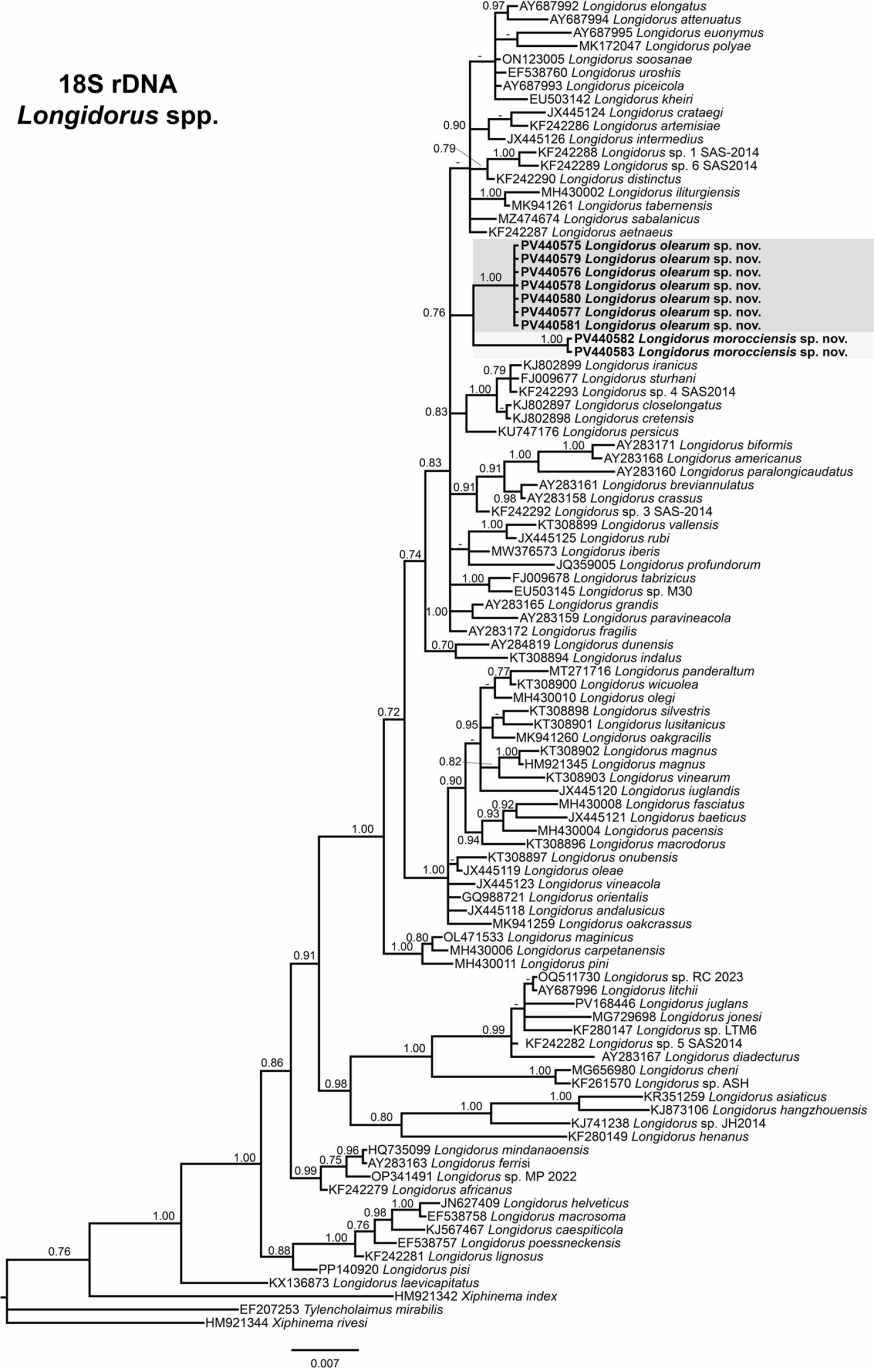
Phylogenetic relationships of *Longidorus* spp. from the Mediterranean Basin within the genus *Longidorus*. Bayesian 50% majority rule consensus tree as inferred from 18 S rRNA gene sequence alignment under the symmetrical model with invariable sites and gamma distribution (SYM + I + G). Posterior probabilities of more than 0.70 are given for appropriate clades. Newly obtained sequences are shown in bold. The scale bar indicates expected changes per site, and colored boxes indicate the clade association within *Longidorus* species analysed in this study

**Fig. 4:**
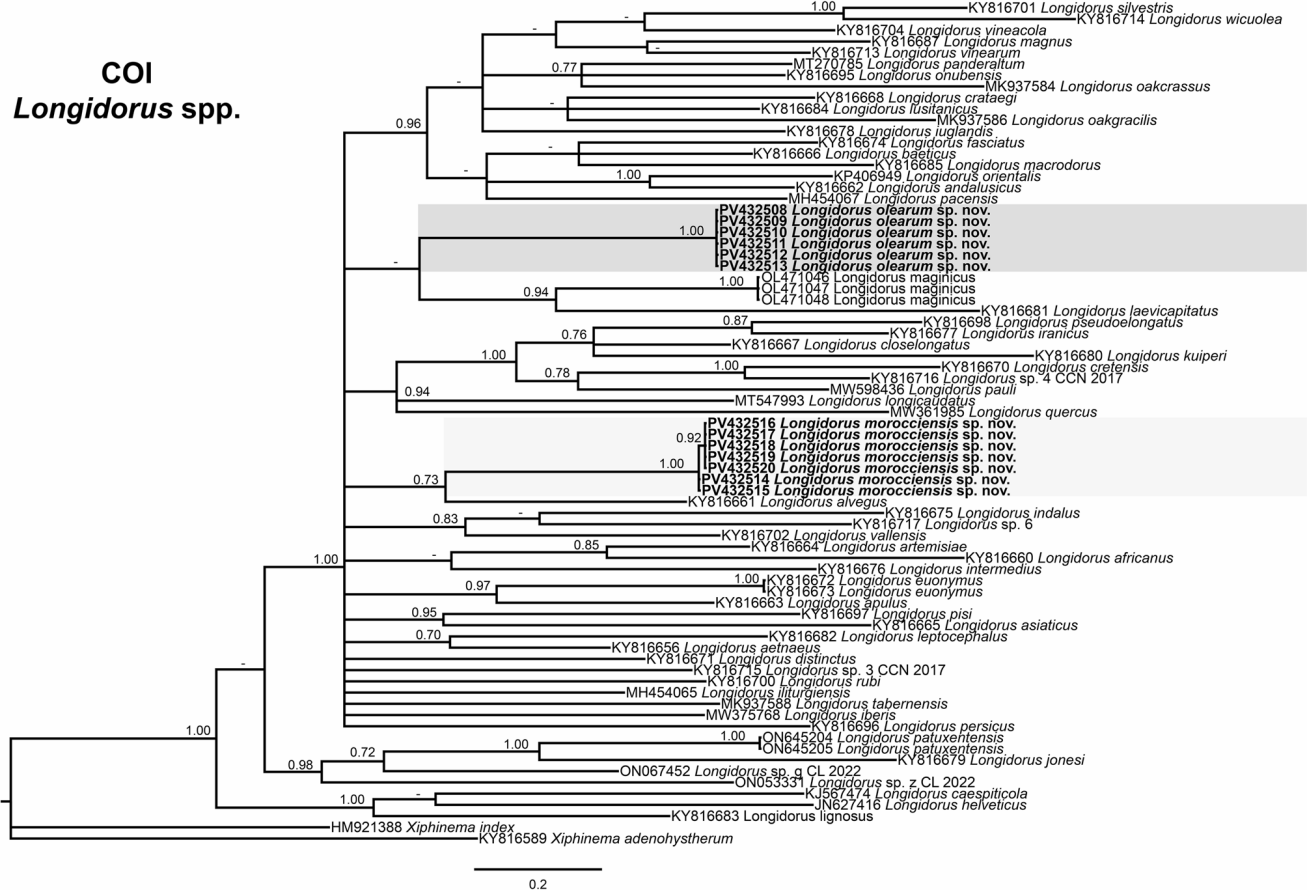
Phylogenetic relationships of *Longidorus* spp. from the Mediterranean Basin within the genus *Longidorus*. Bayesian 50% majority rule consensus tree as inferred from COI mtDNA gene sequence alignment under the under the one-parameter model with invariable sites and gamma distribution model (TPM2uf + I + G). Posterior probabilities of more than 0.70 are given for appropriate clades. Newly obtained sequences in this are shown in bold. The scale bar indicates expected changes per site, and colored boxes indicate the clade association within *Longidorus* species analysed in this study

The phylogenetic relationships among the newly identified needle nematode populations and other *Longidorus* species were inferred using Bayesian inference (BI) based on sequences of the D2–D3 expansion segments of the 28 S rRNA, ITS1 rRNA, partial 18 S rRNA, and mitochondrial COI genes (Figs. 2, 3 and 4).

The D2–D3 alignment (766 bp) included 144 *Longidorus* sequences and three outgroup species: *Tylencholaimus mirabilis* (Bütschli, 1873) [50] de Man, 1876 [17] (AY593027), *Xiphinema index* Thorne & Allen, 1950 [51] (HM921400), and *Xiphinema cretense* Tzortzakakis et al. 2014 [22] (KJ136868). Twenty-five new sequences from this study were incorporated (Fig. 2). The resulting 50% majority-rule consensus tree placed all five species identified here within the main Clade I sensu Subbotin et al. 2014 [52]. The new accessions of *L. olearum* sp. nov. (PV440517-PV440526) and *L. morocciensis* sp. nov. (PV440527-PV440534) formed a well-supported subclade (PP = 0.99), clearly separated from all previously described *Longidorus* species. Populations of *L. magnus*, *L. oakgracilis*, and *L. vineacola* clustered in distinct subclades alongside existing accessions for those species, also within the upper part of Clade I. Overall, Clade I encompassed sequences from 84 *Longidorus* species, predominantly from Mediterranean and European (Palearctic) regions. Clades VIII, V, VI, and IV included 13, eight, eight, and three species, respectively, while Clades II, III, VII, and IX were represented by single species lineages (Fig. 2).

Due to excessive variability, ITS1 rRNA sequences of *L. olearum* sp. nov. (PV448530–PV448531) and *L. morocciensis* sp. nov. (PV448532–PV448539) could not be aligned reliably with available NCBI sequences and were therefore excluded from phylogenetic analysis.

The partial 18 S rRNA gene alignment included 97 sequences, spanning 1715 bp, and three outgroups: *Tylencholaimus mirabilis* (EF207253), *Xiphinema rivesi* (HM921344), and *Xiphinema index* (HM921342) (Fig. 3). *L. olearum* sp. nov. (PV440575–PV440581) and *L. morocciensis* sp. nov. (PV440582–PV440583) clustered together in a subclade with moderate support (PP = 0.76), although they remained clearly distinct from other *Longidorus* species.

Lastly, phylogenetic analysis of the mitochondrial COI gene was based on 73 sequences and a 390 bp alignment, with two outgroup species: *Xiphinema adenohystherum* (KY816589) and *Xiphinema index* (HM921388) (Fig. 4). The phylogenetic resolution was limited: *L. olearum* sp. nov. (PV432508-PV432513) grouped with *L. maginicus* (OL471046-OL471048) in a poorly supported clade (PP = 0.51), while *L. morocciensis* sp. nov. (PV432514-PV432520) clustered with *L. alvegus* (KY816661) with moderate support (PP = 0.73). Both species formed distinct lineages clearly separated from other *Longidorus* species.

### Taxonomic account

Phylum: Nematoda Rudolphi, 1808 [53].

Class: Enoplea Inglis, 1983 [54].

Order: Dorylaimida Pearse, 1942 [55].

Suborder: Dorylaimina Pearse, 1936 [56].

Superfamily: Longidoroidea Khan & Ahmad, 1975 [57].

Family: Longidoridae Thorne, 1935 [58].

 Genus: *Longidorus* Micoletzky, 1922 [59].

### Longidorus olearum sp. nov. (Figs. 5, 6 and 7; Table 2)

**Fig. 5:**
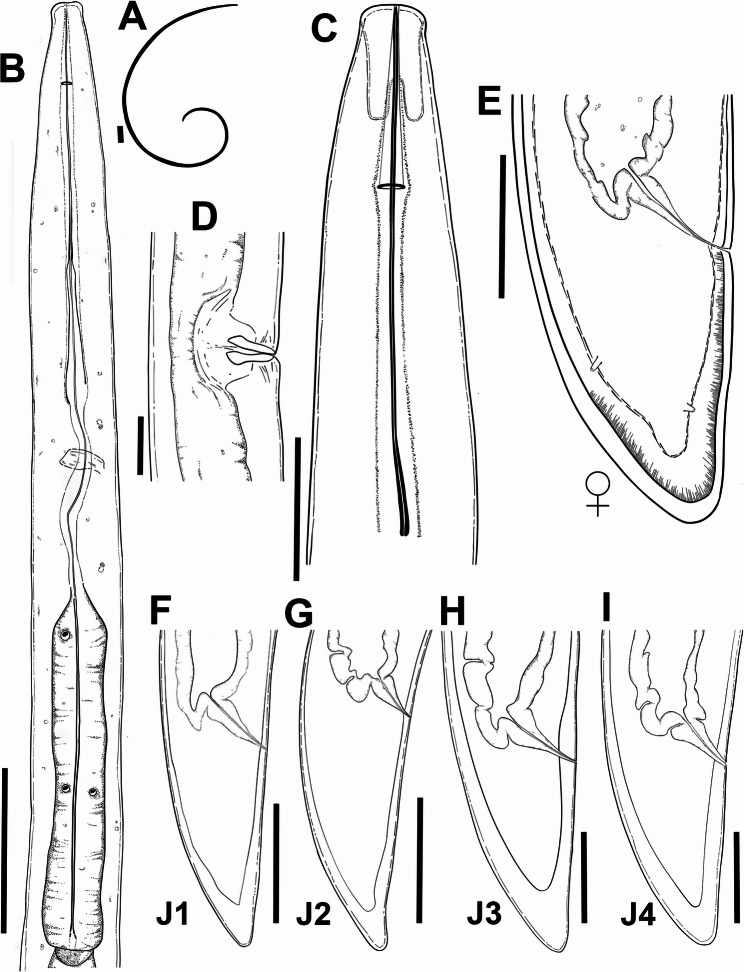
Line drawings of *Longidorus olearum* sp. nov. **A**, whole female; **B**, female pharyngeal region; **C**, lip region showing amphidial fovea; **D**, vulval region; **E**, female tail region; **F**–**I**, tail regions of 1 st, 2nd, 3rd, and 4th stage juveniles (J1, J2, J3 and J4), respectively. Scale bars A = 200 μm; B = 50 μm; C–I = 20 μm

**Fig. 6:**
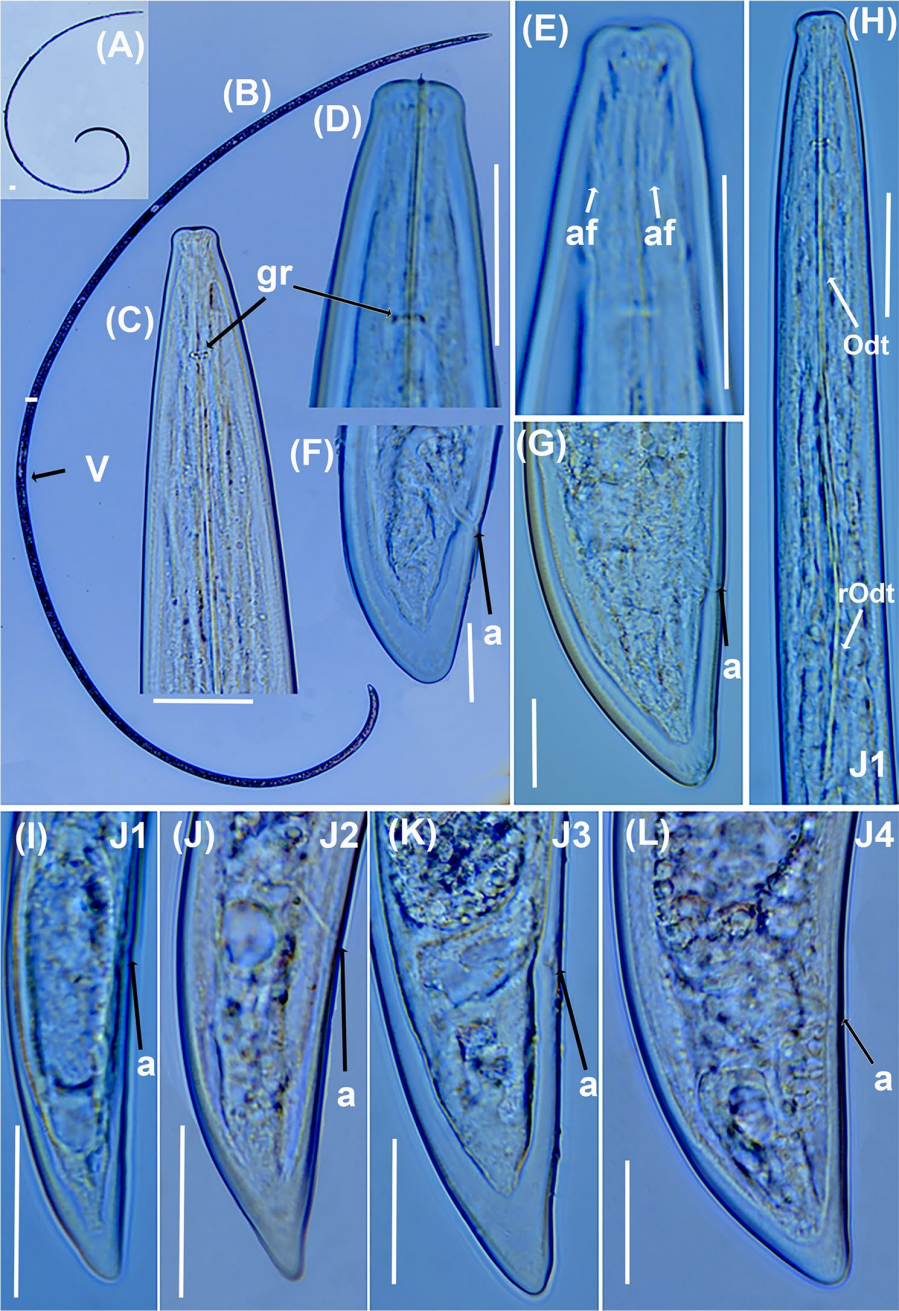
Light micrographs of *Longidorus olearum* sp. nov. **A**, **B**, whole female. **C**-**E**, female lip region showing guiding ring and amphidial fovea (arrowed); **F**, **G**, female tail region. **H**, anterior region of first-stage juvenile showing odontostyle and replacement odontostyle (arrowed); **I**–**L**, tail regions of 1 st, 2nd, 3rd, and 4th stage juveniles (J1, J2, J3 and J4). Abbreviations: a = anus; af = amphidial fovea; gr = guiding ring. Scale bars A = 100 μm; B = 50 μm; C–L = 20 μm

**Fig. 7:**
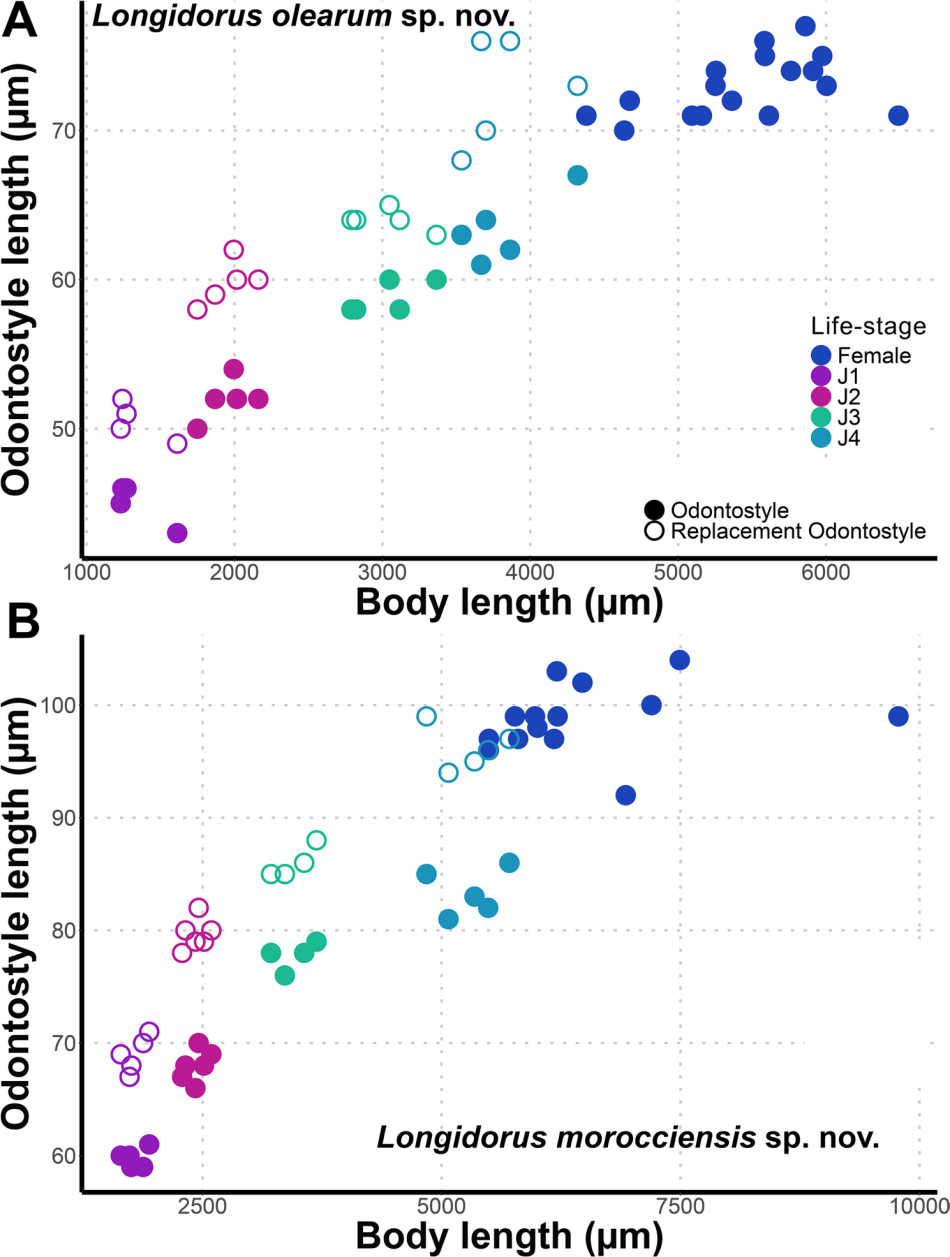
Relationship of body length to length of functional and replacement odontostyle length in all developmental stages from first-stage juveniles (J1) to mature females of: **A**, *Longidorus olearum* sp. nov.; **B**, *Longidorus morocciensis* sp. nov

**Table 2:** Morphometrics of *Longidorus olearum* sp. nov. Holotype and paratypes from Olive (*Olea Europaea* L.) from Sabiote, Jaén province, Spain. All measurements are in µm, except for body length in mm and in the form: mean ± s.d. (range)

Character/ratio^1^	Holotype	Paratypes				
		Female	J1	J2	J3	J4
N		17	4	5	5	5
L (mm)	5.586	5.447 ± 0.554	1.339 ± 0.183	1.958 ± 0.156	3.029 ± 0.235	3.817 ± 0.304
		(4.379–6.489)	(1.231–1.613)	(1.749–2.161)	(2.790–3.366)	(3.535–4.320)
A	133.0	117.4 ± 12.5	67.9 ± 6.1	79.8 ± 8.0	96.3 ± 14.7	105.7 ± 15.9
		(100.5–141.1.5.1)	(59.1–72.6)	(67.3–86.7)	(79.9–116.1.9.1)	(83.4–120.0)
B	11.2	13.2 ± 2.8	6.1 ± 0.5	7.3 ± 0.6	11.1 ± 0.5	12.1 ± 0.4
		(8.8–16.9)	(5.3–6.5)	(6.6–8.0.6.0)	(10.7–11.9)	(11.4–12.4)
C	147.0	135.2 ± 18.9	37.2 ± 3.6	48.4 ± 1.4	69.5 ± 7.6	89.2 ± 7.1
		(98.2–180.3.2.3)	(33.4–41.4)	(46.7–49.9)	(62.0–82.1.0.1)	(80.3–98.2)
c’	1.2	1.3 ± 0.2	2.4 ± 0.4	2.2 ± 0.1	1.9 ± 0.2	1.6 ± 0.1
		(1.1–1.5)	(1.9–2.7)	(2.1–2.2)	(1.7–2.1)	(1.4–1.7)
D	2.5	2.6 ± 0.2	2.3 ± 0.1	2.4 ± 0.1	2.4 ± 0.1	2.4 ± 0.01
		(2.3–2.9)	(2.2–2.4)	(2.2–2.5)	(2.3–2.6)	(2.3–2.5)
d’	1.7	1.8 ± 0.1	1.6 ± 0.01	1.7 ± 0.1	1.7 ± 0.01	1.7 ± 0.01
		(1.6–2.0.6.0)	(1.5–1.6)	(1.6–1.8)	(1.6–1.7)	(1.6–1.7)
V or T	51.4	51.7 ± 0.7	-	-	-	-
		(50.8–53.4)				
G_1_	6.8	7.4 ± 1.1	-	-	-	-
		(5.3–9.3)				
G_2_	6.8	7.3 ± 1.1	-	-	-	-
		(5.3–9.2)				
Odontostyle	75.0	72.9 ± 2.1	45.0 ± 1.4	52.0 ± 1.4	58.8 ± 1.1	63.4 ± 2.3
		(70.0–77.0)	(43.0–46.0)	(50.0–54.0)	(58.0–60.0)	(61.0–67.0)
Odontophore	40.0	40.6 ± 2.4	32.5 ± 1.3	32.8 ± 2.3	37.2 ± 1.3	38.6 ± 3.6
		(36.0–44.0)	(31.0–34.0)	(30.0–36.0)	(36.0–39.0)	(34.0–43.0)
Total stylet	115.0	113.5 ± 3.3	77.5 ± 2.5	84.8 ± 3.3	96.0 ± 1.9	102.0 ± 4.0
		(107.0–118.0.0.0)	(74.0–80.0)	(80.0–88.0)	(94.0–98.0)	(98.0–106.0.0.0)
Replacement odontostyle	-	-	50.5 ± 1.3	59.8 ± 1.5	64.0 ± 0.7	72.6 ± 3.6
			(49.0–52.0)	(58.0–62.0)	(63.0–65.0)	(68.0–76.0)
Lip region diam.	10.0	10.0 ± 0.5	8.1 ± 0.3	8.3 ± 0.4	8.9 ± 0.2	10.4 ± 0.5
		(9.0–11.0)	(8.0–8.5.0.5)	(8.0–9.0)	(8.5–9.0.5.0)	(10.0–11.0)
Oral aperture to guiding ring	25.0	26.0 ± 1.2	18.9 ± 0.6	19.8 ± 0.3	21.7 ± 1.3	22.8 ± 1.4
		(25.0–29.0)	(18.0–19.5.0.5)	(19.5–20.0)	(19.5–23.0)	(21.5–25.0)
Max. body diam.	41.0	46.0 ± 5.9	19.8 ± 2.4	24.6 ± 1.1	32.0 ± 5.2	36.6 ± 4.7
		(34.0–55.0)	(17.5–22.5)	(23.0–26.0)	(27.0–39.0)	(31.0–44.0)
Tail length	42.0	39.9 ± 3.5	36.0 ± 3.2	40.4 ± 2.5	43.7 ± 1.9	42.8 ± 1.3
		(36.0–47.0)	(32.0–39.0)	(37.0–44.0)	(41.0–46.0)	(41.0–44.0)
J	13.0	10.1 ± 1.4	7.0 ± 0.7	8.2 ± 0.8	10.8 ± 1.3	9.4 ± 0.5
		(8.0–13.0)	(6.5–8.0.5.0)	(7.5–9.5)	(9.0–12.0)	(9.0–10.0)

^1^ as defined in Jairajpuri & Ahmad [31]. a, body length/maximum body width; b, body length/pharyngeal length; c, body length/tail length; c', tail length/body width at anus; V (distance from anterior end to vulva/body length) x 100; d anterior to guiding ring/body diam. at lip region, d’ body diam. at guiding ring/body diam. at lip region, J hyaline tail region length

#### Zoobank

urn: lsid: zoobank.org: pub: 97CF6AD6-281D-4F93-BDF8-223342B0CBAA.

#### Holotype

Adult female was extracted from a soil sample collected from the rhizosphere of cultivated olive (*Olea europaea* subsp. *europaea* (Mill.) Lehr), in Sabiote, Jaén province, southern Spain (38°06’19"N latitude 3°13’36"W485 longitude) by J. Martín Barbarroja and G. León Ropero, mounted in pure glycerine and deposited in the Nematode Collection of the Institute for Sustainable Agriculture, CSIC, Córdoba, Spain (slide number SO1_L1).

#### Paratypes

Seventeen female paratypes and four or five specimens from each juvenile-stage (J1–J4) were collected simultaneously with the holotype from the type locality by R. Salazar-García and A. Archidona-Yuste, mounted in pure glycerine, and deposited in the Nematode Collection of the Institute for Sustainable Agriculture, CSIC, Córdoba, Spain (slide numbers SO1_L2 to SO1_L11). Additionally, one female paratype is deposited on slide number T-8212p in the USDA Nematode Collection, Beltsville, MD, USA.

#### Etymology

The species epithet is the Latin term *olearum* = belonging to, or corresponding to olives, as type material was found in an olive grove.

#### Diagnosis and relationships

*Longidorus olearum* sp. nov. is a parthenogenetic species characterized by a moderately long body (4.4–6.5 mm); a lip region that is anteriorly flattened and slightly set off from the rest of the body by a shallow depression (9–11 μm wide); a symmetrically bilobed amphidial fovea; a relatively short odontostyle (70–77 μm); and a female tail that is bluntly conoid with a rounded terminus. According to the polytomous key by Chen et al. 1997 [60], *L. olearum* sp. nov. is closely related to *L. unedoi* Arias et al. 1986 [61], *L. attenuatus*, and *L. indalus* Archidona-Yuste et al. 2016 [28], from which it can be distinguished based on a combination of morphological characters, and most notably, the absence of males.

*Longidorus olearum* sp. nov. differs from *L. unedoi* by having a lower a ratio (100.5–141.1 vs. 121–165.5), a lower b ratio (8.8–16.9 vs. 13–19), a smaller c′ ratio (1.1–1.5 vs. 1.4–2.0), a lower V value (50.8–53.4 vs. 52–58), and a significantly longer odontostyle (70–77 vs. 52–64 μm). It can be distinguished from *L. attenuatus* by its shorter body (4.4–6.5 vs. 5.2–7.5 mm), smaller c′ ratio (1.1–1.5 vs. 1.5–1.9), and higher a ratio (100.5–141.1 vs. 109–210). Compared to *L. indalus*, *L. olearum* sp. nov. presents a smaller c′ ratio and higher V value (1.1–1.5 vs. 1.9–2.9 and 50.8–53.4 vs. 45.5–50.0, respectively), as well as a longer odontostyle (70–77 vs. 54–60 μm).

According to the polytomous key by Chen et al. 1997 [60], the diagnostic codes for *L. olearum* sp. nov. are: A2-B1-C2-D3-E2-F3-G2(3)-H2-I1 (with codes in parentheses indicating exceptions).

#### Description

##### Female

Body moderately long and thin, (4.4–6.5) mm, when heat relaxed it forms a close C to open spiral. Cuticle appears smooth and thin, 2 μm thick at mid-body and 8.0–13.0 μm thick at tail tip. Lip region anteriorly flattened and slightly offset by a depression. Amphidial fovea pouchlike and symmetrically bilobed, opening not visible. Lateral cord 11 (10–12) µm wide at mid-body, or 21.8–32.4% of corresponding body diameter. Guiding ring located 2.6 ± 0.1 (2.3–2.9) times lip region diameter from anterior end (Table 2). Odontostyle relatively long and narrow, 1.8 ± 0.1 (1.6–2.1) times as long as odontophore. Odontophore well developed. Basal bulb (104–134) µm long and (16–24) µm wide. Dorsal pharyngeal gland nucleus (DN) and ventro-sublateral pair of nuclei (SN) situated at 12.6 ± 3.3 (7.8–15.8)% and 47.7 ± 5.0 (42.3–55.3) % of distance from anterior end of pharyngeal bulb, respectively. Glandularium 94.7 ± 6.0 (86.0–101.0) µm long. Cardia conoid rounded, 8.0–9.0 μm long. Reproductive system with both genital branches equally developed (Table 2). Ovaries straight and stable in length between the two branches, (57–63) µm. Vulva appearing as a transverse slit positioned around the middle of the body. Vagina oriented perpendicular to the body axis, measuring 12.4 (10.0–15.0) µm in length, and surrounded by well-developed muscles. Uteri 136 (135–137) µm long, sperm cells not detected in any of the females examined. Rectum (0.4–0.7) times as long as anal body diameter, prerectum (15.9–18.0) times as long as anal body diameter. Tail bluntly conoid, dorsally convex, ventrally almost flat, with a rounded terminus, and presents two or three pairs of caudal pores.

##### Male

Not found.

##### Juveniles

Morphologically similar to adults, but smaller. The four-juvenile life-stages were distinguishable by relative length of functional and replacement odontostyle and body length (Fig. 6). The juveniles’ tails showed differences depending on their life-stage, c′ is higher in younger juveniles and the tail is shortened as the life-stage juveniles grow into adults. The first-stage juvenile (J1) exhibits a slightly rounded yet relatively elongated tail, with a c′ ratio ranging from 1.9 to 2.4. It possesses a functional odontostyle measuring 45 μm, which is approximately 39% shorter than that of the adult (Fig. 7). In J1, the tip of the replacement odontostyle is located near the base of the functional odontostyle. In contrast, in the three subsequent juvenile stages, the replacement odontostyle is positioned further away from the functional odontostyle.

**Fig. 8:**
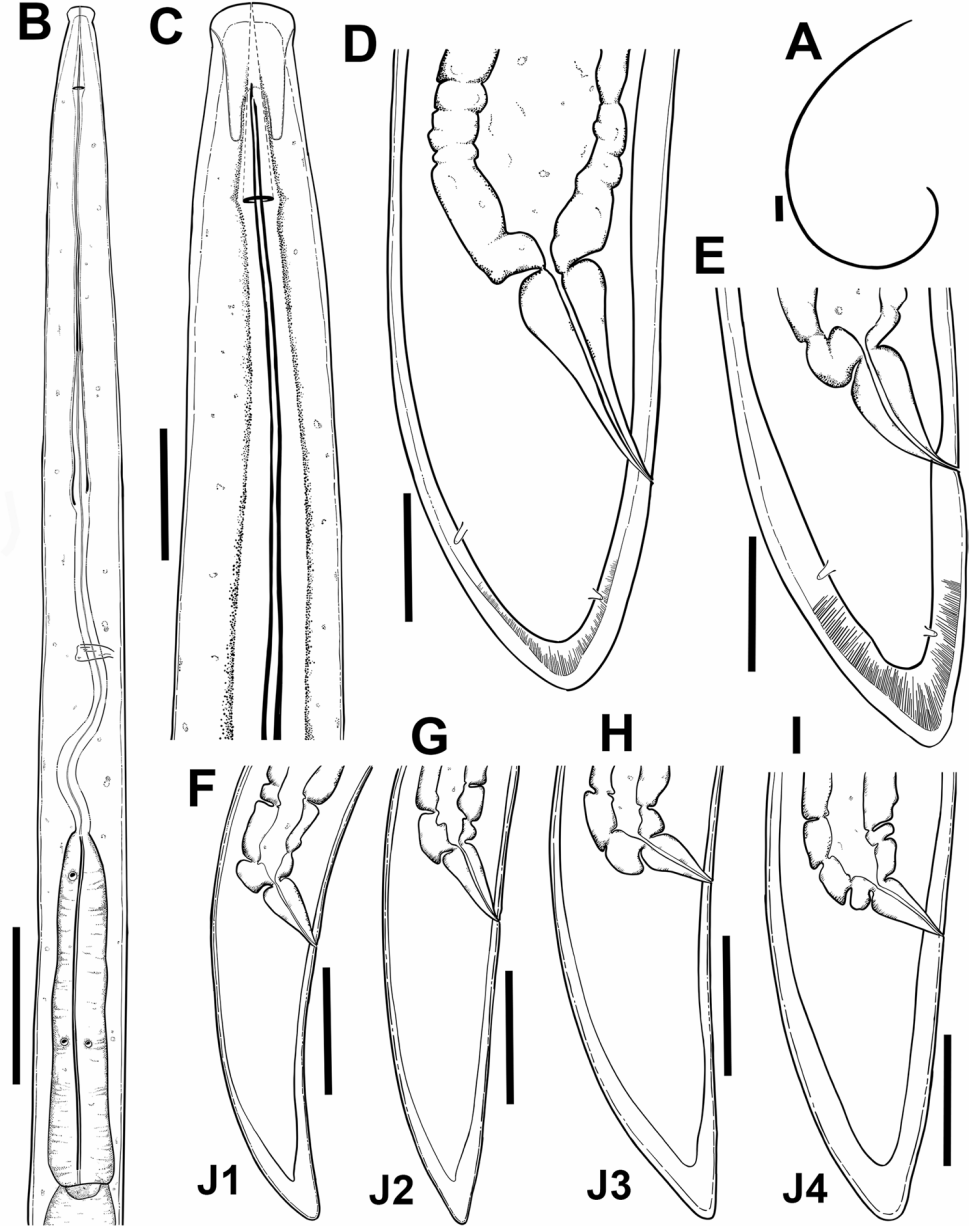
Line drawings of *Longidorus morocciensis* sp. nov. **A**, whole female; **B**, female pharyngeal region; **C**, lip region showing amphidial fovea; **D**, **E**, female tail region; **F**-**I**, tail regions of 1 st, 2nd, 3rd, and 4th stage juveniles (J1, J2, J3 and J4), respectively. Scale bars A = 200 μm; B = 50 μm; C-I = 20 μm

### Longidorus morocciensis sp. nov. (Figs. 7, 8 and 9; Table 3.)

**Fig. 9:**
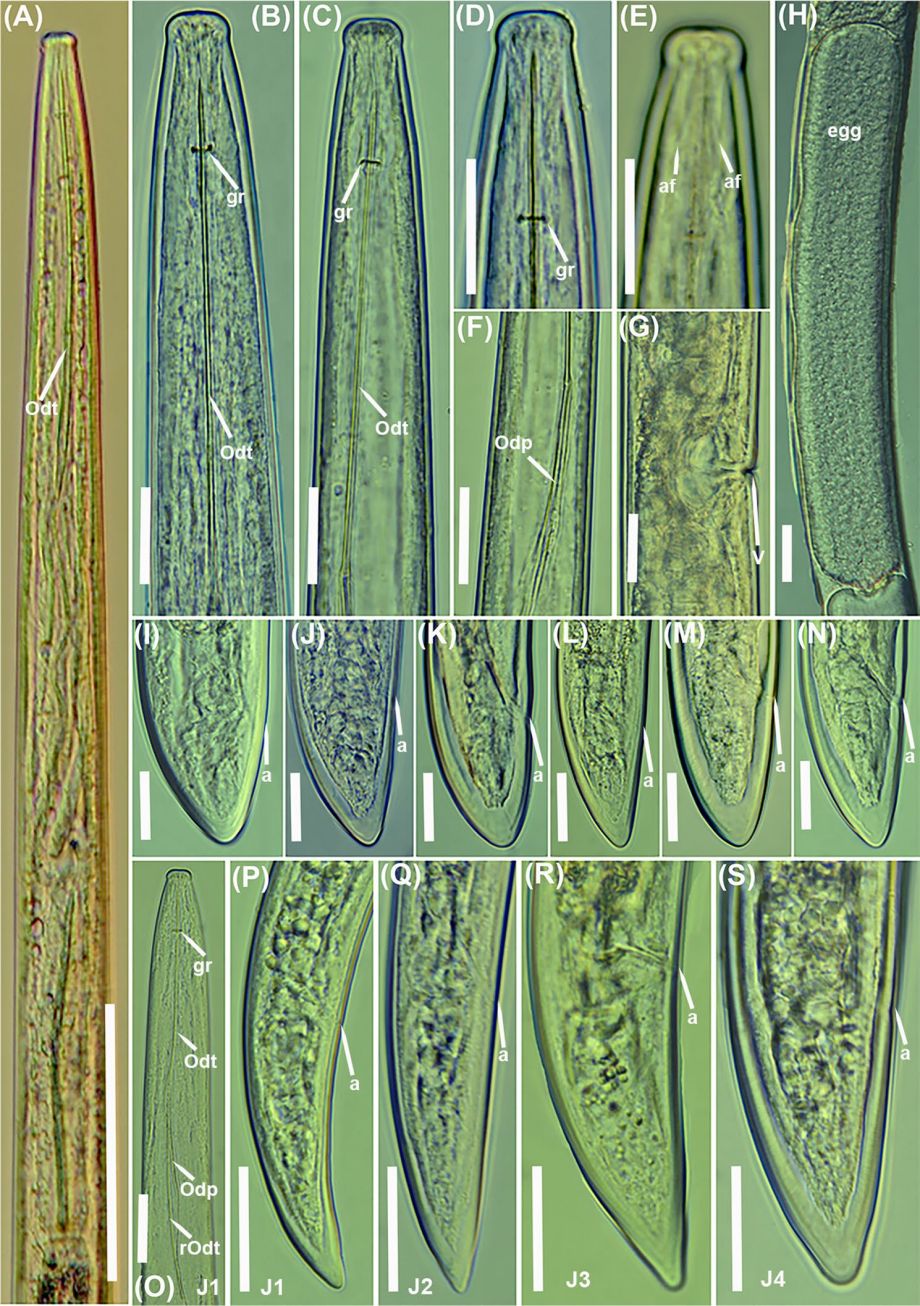
Light micrographs of *Longidorus morocciensis* sp. nov. **A**, female neck region (odontostyle arrowed); **B**-**E**, female lip region showing odontostyle, guiding ring and amphidial fovea (arrowed); **F**, detail of female odontophore (arrowed); **G**, vulval region; **H**, detail of an egg inside the body; **I**–**N**, female tail region; **O**, anterior region of first-stage juvenile showing odontostyle, odontophore and replacement odontostyle (arrowed); **P**-**S**, tail regions of 1 st, 2nd, 3rd, and 4th stage juveniles (J1, J2, J3 and J4). Abbreviations: a = anus; af = amphidial fovea; egg = egg; gr = guiding ring; Odt = odontostyle; Odp = odontophore, rOdt = replacement odontostyle; V = vulva. Scale bars A = 100 μm; B–S = 20 μm

**Table 3.** Morphometrics of *Longidorus morocciensis*sp. nov. holotype and paratypes from wild olive (*Olea europaea* subsp. *maroccana* (Greuter and Burdet) Vargas *et al*.), from Meknès, Fez-Meknès region, Morocco. All measurements are in µm, except for body length in mm and in the form: mean ± s.d. (range)

Paratypes						
Character/ratio^1^	Holotype	Female	J1	J2	J3	J4
N		14	5	6	4	5
L (mm)	6.780	6.286 ± 0.616	1.791 ± 0.119	2.433 ± 0.116	3.458 ± 0.211	5.292 ± 0.341
		(5.495-7.492)	(1.644-1.942)	(2.286-2.592)	(3.216-3.692)	(4.843-5.710)
A	132.9	136.7 ± 10.0	73.0 ± 8.3	95.2 ± 4.8	113.0 ± 9.8	114.9 ± 3.5
		(121.7-154.1)	(65.0-82.2)	(89.4-100.9)	(103.7-122.9)	(110.1-119.0)
B	15.0	15.6 ± 1.2	6.8 ± 0.3	7.8 ± 0.3	10.4 ± 0.4	16.6 ± 1.0
		(13.7-17.2)	(6.5-7.2)	(7.2-8.2)	(9.9-10.7)	(14.8-17.4)
C	165.4	157.3 ± 22.8	39.8 ± 2.9	51.5 ± 3.8	69.2 ± 2.4	116.1 ± 8.0
		(134.1-200.8)	(37.3-43.2)	(46.7-55.9)	(67.0-71.5)	(105.3-126.9)
c'	1.2	1.3 ± 0.1	2.9 ± 0.2	2.5 ± 0.2	2.3 ± 0.1	1.7 ± 0.03
		(1.1-1.5)	(2.8-3.3)	(2.3-2.8)	(2.2-2.4)	(1.6-1.7)
D	2.6	2.5 ± 0.2	2.4 ± 0.1	2.3 ± 0.1	2.4 ± 0.1	2.5 ± 0.08
		(2.0-2.7)	(2.3-2.5)	(2.2-2.4)	(2.3-2.4)	(2.5-2.6)
d'	1.7	1.6 ± 0.1	1.7 ± 0.1	1.5 ± 0.04	1.5 ± 0.1	1.6 ± 0.04
		(1.5-1.8)	(1.6-1.8)	(1.5-1.6)	(1.4-1.6)	(1.5-1.6)
V or T	51.4	50.6 ± 1.0	-	-	-	-
		(49.0-52.7)				
G_1_	8.1	8.5 ± 3.3 (5.5-16.8)	-	-	-	-
G_2_	8.1	8.2 ± 2.8 (5.4-15.1)	-	-	-	-
Odontostyle	99.0	98.7 ± 3.1	58.9 ± 0.8	68.0 ± 1.4	77.8 ± 1.3	83.4 ± 2.1
		(92.0-104.0)	(59.0-61.0)	(66.0-70.0)	(76.0-79.0)	(81.0-86.0)
Odontophore	55.0	54.5 ± 2.8	38.2 ± 0.8	42.3 ± 1.4	46.3 ± 1.7	50.6 ± 1.9
		(50.0-59.0)	(37.0-39.0)	(40.0-44.0)	(44.0-48.0)	(49.0-54.0)
Total stylet	154.0	153.4 ± 4.3	98.0 ± 1.2	110.3 ± 2.3	124.0 ± 2.4	134.0 ± 3.5
		(146.0-161.0)	(97.0-100.0)	(107.0-113.0)	(122.0-127.0)	(130.0-139.0)
Replacement odontostyle	-	-	69.0 ± 1.6	79.6 ± 1.4	86.0 ± 1.4	96.2 ± 1.9
			(67.0-71.0)	(78.0-82.0)	(85.0-88.0)	(94.0-99.0)
Lip region diam.	12.5	11.8 ± 0.5	8.2 ± 0.3	9.8 ± 0.3	10.8 ± 0.5	10.9 ± 0.2
		(10.5-12.5)	(8.0-8.5)	(9.5-10.0)	(10.0-11.0)	(10.5-11.0)
Oral aperture to guiding ring	32.0	29.0 ± 2.4	19.9 ± 0.2	22.6 ± 0.5	25.3 ± 1.0	27.3 ± 1.0
		(24.0-32.0)	(19.5-20.0)	(22.0-23.0)	(24.0-26.0)	(26.5-29.0)
Max. body diam.	51.0	46.1 ± 4.4	24.8 ± 3.3	25.7 ± 2.4	30.8 ± 3.1	46.0 ± 1.6
		(39.0-54.0)	(20.0-28.0)	(23.0-29.0)	(28.0-35.0)	(44.0-48.0)
Tail length	41.0	40.3 ± 3.8	45.0 ± 1.2	47.3 ± 2.1	50.0 ± 2.9	45.6 ± 0.5
		(33.0-46.0)	(44.0-47.0)	(45.0-50.0)	(47.0-53.0)	(45.0-46.0)
J	11.0	10.8 ± 1.2	6.7 ± 0.4	10.0 ± 1.0	8.0 ± 0.8	10.3 ± 1.0
		(9.0-12.0)	(6.0-7.0)	(9.0-11.0)	(7.0-9.0)	(9.5-12.0)

^1^ Abbreviations as defined in Jairajpuri & Ahmad [31]. a, body length/maximum body width; b, body length/pharyngeal length; c, body length/tail length; c', tail length/body width at anus; V (distance from anterior end to vulva/body length) x 100; d anterior to guiding ring/body diam. at lip region, d’ body diam. at guiding ring/body diam. at lip region, J hyaline tail region length

#### Zoobank

urn: lsid: zoobank.org: act: DC765E56-C6A6-4C12-BBE9-29D96D6558DF.

#### Holotype

Adult female was extracted from a soil sample collected of wild olive (*Olea europaea* subsp. *maroccana* (Greuter and Burdet) Vargas et al.), from Meknès, Fez-Meknès region, Morocco (33°47’43"N latitude 5°39’55"W longitude, 473 m a.s.l.) by A. Bajoub, mounted in pure glycerine and deposited in the Nematode Collection of the Institute for Sustainable Agriculture, CSIC, Córdoba, Spain (slide number ZN_4_3_L1).

#### Paratypes

Fourteen female paratypes, along with five J1, six J2, four J3, and five J4 paratypes, were collected simultaneously with the holotype from the type locality by A. Bajoub. These were mounted in pure glycerine and deposited in the Nematode Collection of the Institute for Sustainable Agriculture, CSIC, Córdoba, Spain (slide numbers ZN_4_3_L2 to ZN_4_3_L13). Additionally, one female paratype was deposited in the USDA Nematode Collection (slide T-8213p).

#### Etymology

The species epithet refers to the country where the new species was first detected, Morocco.

#### Diagnosis and relationships

*Longidorus morocciensis* sp. nov. is a parthenogenetic species characterized by a long body (5.5–7.5 mm); lip region rounded, slightly separated from the rest of body by depression, 10.5–12.5 µm wide, amphidial fovea symmetrically bilobed; long odontostyle (92–104 µm), and female tail conoid-rounded, dorsally convex, ventrally nearly straight, with narrow, bluntly rounded tip. According to the polytomous key by Chen et al. 1997 [60] *L. morocciensis* sp. nov. is closely related to *L. protae*, *L. glycines* and *L. iranicus*, from which it can be differentiated by combining the characters presented below. *Longidorus morocciensis* sp. nov. differs from *L. protae* by having a longer odontostyle (92–104 vs 73–83) µm; a longer female tail (33–46 vs 32–37) µm; and a longer distance from guiding ring to oral aperture (24–32 vs 18–21 µm). From *L. glycines* differs by a longer distance from guiding ring to oral aperture (24–32 vs. 22–26 µm); narrower lip region (10.5–12.5 *vs*. 20.3–23.3 µm); shorter body (4.4–6.5 vs. 5.2–7.5 mm); a smaller a ratio (121.7–154.1 vs. 113.4–188.2); a higher c′ ratio (1.1–1.5 vs. 0.9–1.3); and the presence of male (absent *vs*. present). Furthermore, *L. morocciensis* sp. nov. is distinguishable from *L. iranicus* by lip region shape (lip region rounded, slightly separated from the rest of body by depression vs narrow and continuous with the rest of body); a higher a ratio (121.7–154.1 *vs*. 92–115); a higher female c’ ratio (1.1–1.5 *vs*. 0.8–1.0); and a higher first-stage juvenile c′ ratio (2.8–3.3 *vs*. 2.3–2.8). According to the polytomous key by Chen et al. 1997 [60], *L. morocciensis* sp. nov. codes are (codes in parentheses are exception): A3-B1(2)-C2(3)-D3-E2-F3(4)-G3-H2-I1.

#### Description

##### Female

Body rather long and thin, 6.3 (5.5–7.5) mm, when heat relaxed it forms a close C to open spiral. Cuticle appears smooth and thin, 2.5–3.0 μm thick along the centre of the body and 9.0–12.0 μm thick at tail tip. Lip region rounded and slightly offset by depression. Amphidial fovea pouchlike and symmetrically bilobed, opening not visible. Lateral cord 10.5 (10.0–11.0) µm wide at mid-body. Single stylet guiding ring, located 2.5 ± 0.2 (2.0–2.7) times lip region diameter from anterior end (Table 3.). Odontostyle relatively long and narrow, 1.8 ± 0.1 (1.7–2.1) times as long as odontophore. Odontophore well developed. Basal bulb 134.4 ± 6.7 (124.0–143.0) µm long and 19.0 ± 1.6 (18.0–23.0) µm wide. Dorsal pharyngeal gland nucleus (DN) and ventro-sublateral pair of nuclei (SN) situated at 14.1 ± 1.8 (12.8–15.3) % and 51.6 ± 2.8 (49.6–53.6) % of distance from anterior end of pharyngeal bulb, respectively. Glandularium 118.8 ± 4.4 (110.0–125.0) µm long. Cardia conoid rounded, 8.0–10.0 μm long. Reproductive system with both genital branches equally developed. Ovaries straight and constant in length between the two branches, (104–118) µm. Vulva appearing as a transverse slit positioned around the middle of the body. Vagina oriented perpendicular to the body axis, measuring (10–16) µm in length, and surrounded by well-developed muscles. Rectum (0.7–1.1) times as long as anal body diameter. Tail conoid rounded, dorsally convex, ventrally almost straight to slightly convex, with narrow bluntly rounded tip, and presenting two pairs of caudal pores.

##### Male

Not found.

##### Juveniles

Morphologically similar to adults, but smaller. The four-juvenile life-stages were distinguishable by relative length of functional and replacement odontostyle and body length (Fig. 7). Juveniles’ tails showed life-stage-dependent differences; c′ is higher in younger juveniles and the tail is shortened as the juveniles grow into adults. First stage-juvenile (J1) present a conoid but relatively long tail, c′ ratio between 2.8 and 3.3, a functional odontostyle of 60.0 μm which is 60.8% smaller than the adult size (Fig. 7).

### New populations from known species: Longidorus magnus, L. oakgracilis, and L. vineacola

**Fig. 10:**
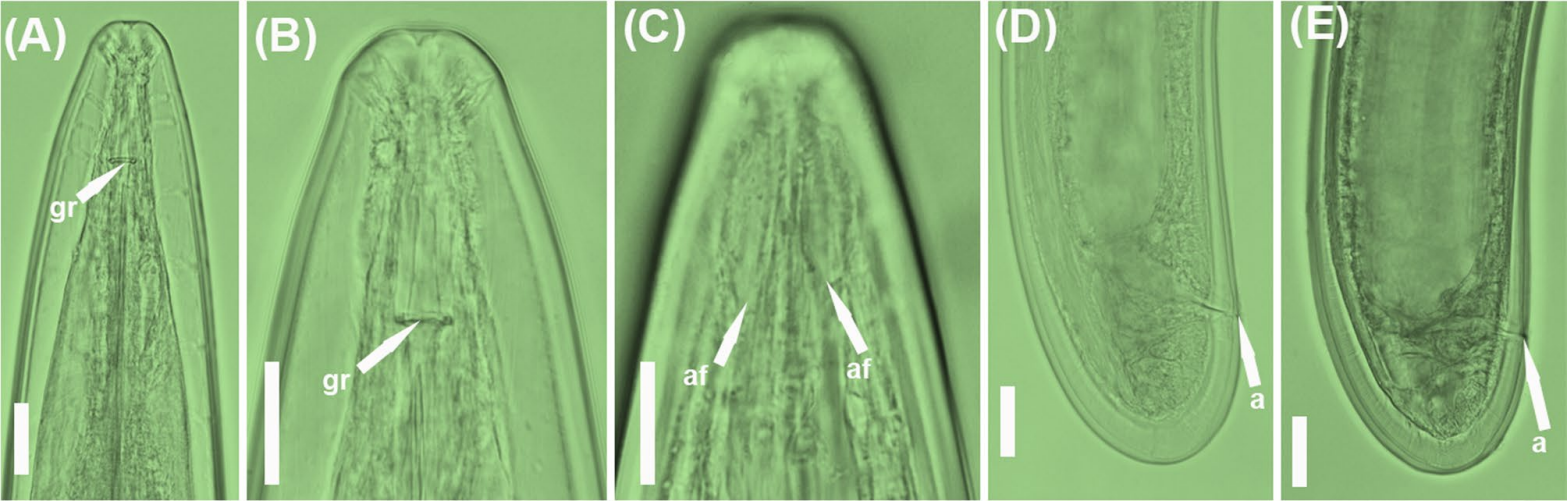
Light micrographs of *Longidorus magnus* Lamberti et al. 1982 [48].**A**–**C**, female lip regions showing guiding ring and amphidial fovea (arrowed); **D**,**E**, female tail region showing anus (arrowed). Abbreviations: a = anus; af = amphidial fovea; gr = guiding ring. Scale bars = 20 μm

**Fig. 11:**
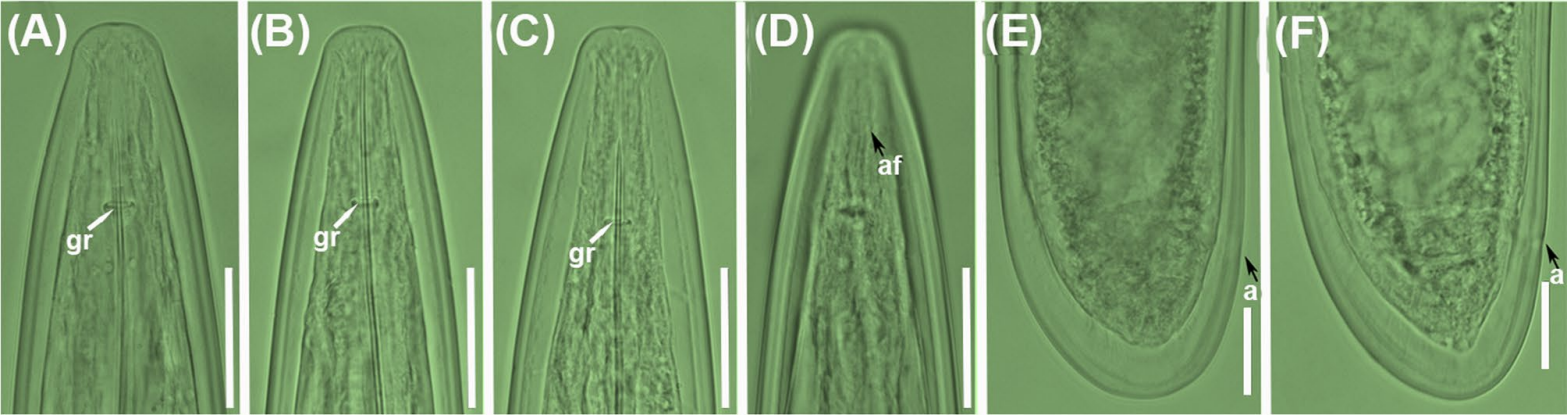
Light micrographs of *Longidorus oakgracilis* Cai et al. 2020b [49].**A**-**D**, female lip regions showing guiding ring and amphidial fovea (arrowed); **E**,**F**, female tail region showing anus (arrowed). Abbreviations: a = anus; af = amphidial fovea; gr = guiding ring. Scale bars = 20 μm

**Fig. 12:**
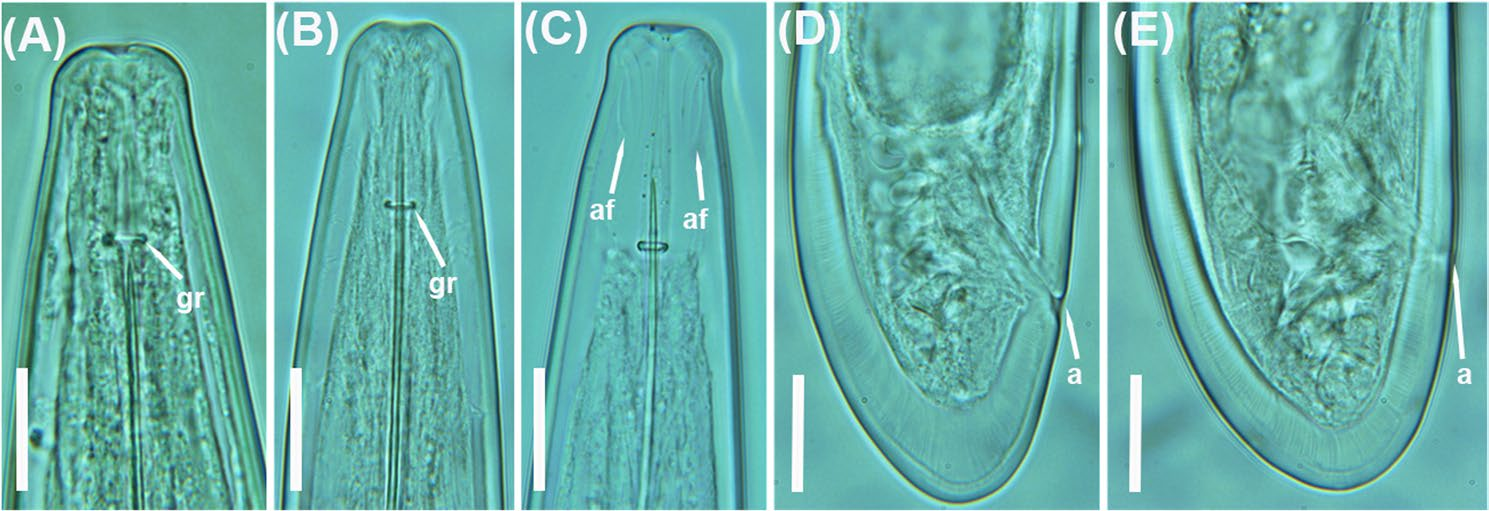
Light micrographs of*Longidorus vineacola* Sturhan & Weischer, 1954 [47].**A**-**C**, female lip regions showing guiding ring and amphidial fovea (arrowed); **D**,**E**, female tail region showing anus (arrowed). Abbreviations: a = anus; af = amphidial fovea; gr = guiding ring. Scale bars = 20 μm

Morphological, morphometric, and molecular data (see below) from the seven populations identified as *L. magnus*, *L. oakgracilis*, and *L. vineacola* (Tables 1 and 4; Figs. 10, 11 and 12) have been previously reported in studies on needle nematodes in Spain [28, 39]. Accordingly, only brief remarks and the D2–D3 expansion segments of the 28 S rRNA gene are presented herein (Fig. 2).

**Table 4:** Morphometrics of *Longidorus Magnus* Lamberti et al. 1982 [48], *L. oakgracilis* Cai et al. 2020 [49], and *L. vineacola* Sturhan & Weischer, 1954 [47] from cultivated Olive (*Olea Europaea* ssp. *Europaea*) and globe-fruited Retama (*Retama sphaerocarpa*) at Portugal and Spain. All measurements are in µm, except for body length in mm and in the form: mean ± s.d. (range)

Character/ratio^1^	Longidorus magnus				L. oakgracilis		L. vineacola
	Female	Female	Female	Female	Female	Female	Female
Sample code	SO07	SO09	SO12	SO14	SO20	ARR2	SO05
n	2	1	1	1	2	1	2
L (mm)	(10.833, 11.316)	9.288	10.120	11.404	(6.420, 6.779)	6599	(9.432, 9.354)
a	(79.0, 82.3)	66.3	71.3	81.4	(71.3, 73.7)	72.5	(147.4, 148.5)
b	(20.0, 20.3)	14.8	16.3	17.0	(14.4, 15.0)	14.7	(19.5, 20.0)
c	(173.6, 187.1)	164.1	168.7	193.2	(173.5, 176.1)	174.8	(269.5, 292.3)
c’	(0.7, 0.7)	0.6	0.7	0.7	(0.7, 0.7)	0.7	(0.7, 0.8)
d	(1.8, 2.0)	2.1	2.0	2.0	(1.8, 1.9)	1.9	(1.6, 1.7)
d’	(1.6, 1.9)	1.8	1.8	1.8	(1.6, 1.6)	1.6	(1.3, 1.3)
V or T	(50.3, 52.7)	51.7	51.0	53.0	(52.3, 53.3)	52.8	(51.0, 51.5)
G_1_	(5.7, 6.2)	6.5	7.1	5.8	(4.7, 5.2)	5.0	(6.9, 7.9)
G_2_	(5.6, 6.0)	6.4	7.0	5.8	(4.6, 5.0)	4.8	(6.9, 7.2)
Odontostyle	(130.0, 135.0)	132.0	129.0	132.0	(95.0, 96.0)	95.5	(91.0, 94.0)
Odontophore	(84.0, 92.0)	82.0	83.0	85.0	(58.0, 60.0)	59	(54.0, 58.0)
Total stylet	(219.0, 222.0)	214.0	212.0	217.0	(153.0, 156.0)	154.5	(145.0, 152.0)
Lip region diam.	(23.0, 27.0)	23.0	24.0	24.0	(16.0, 17.0)	16.5	(18.5, 19.0)
Oral aperture to guiding ring	(46.0, 48.0)	42.0	43.0	44.0	(30.0, 31.0)	30.5	(30.0, 31.0)
Max. body diam.	(131.0, 143.0)	140.0	142.0	140.0	(90.0, 92.0)	91.0	(63.0, 64.0)
Tail length	(58.0, 65.0)	56.5	60	59	(37.0, 38.5)	38.0	(32.0, 35.0)
J	(14.0, 17.0)	16.5	15.0	17.0	(12.0, 14.0)	13.0	(12.0, 13.0)

^1^ Abbreviations as defined in Jairajpuri & Ahmad [31]. a, body length/maximum body width; b, body length/pharyngeal length; c, body length/tail length; c’, tail length/body width at anus; V (distance from anterior end to vulva/body length) x 100; d anterior to guiding ring/body diam. at lip region, d’ body diam. at guiding ring/body diam. at lip region, J hyaline tail region length

The morphology and measurements of four *L. magnus* populations collected from various localities in Spain align closely with the original description and more recent reports of this species [28], thereby extending its known distribution in olive groves (Table 4; Fig. 10). Likewise, the two *L. oakgracilis* populations (Table 4; Fig. 11) recovered from olive groves in two Portuguese localities are consistent with the original description of this species from natural vegetation in Spain [49]. To our knowledge, this is the first report of *L. oakgracilis* in Portugal, suggesting a broader distribution in cultivated olive within the Iberian Peninsula.

Finally, the morphological and morphometric features of the single *L. vineacola* population from Chiclana de Segura, southern Spain, agree with previous records of the species in the region [28] and further expand its geographic range (Table 4; Fig.12). Notably, *L. vineacola* was detected at high population densities in globe-fruited retama (*Retama sphaerocarpa*), indicating that this may be a particularly suitable host for the species.

## Discussion

The primary objective of the present study was to clarify the biodiversity and molecular phylogeny of needle nematodes of the genus *Longidorus* detected in Mediterranean olive groves managed under organic and conventional regimes, as well as in nearby patches of natural vegetation in Greece, Italy, Morocco, Portugal, and Spain. In total, nine*Longidorus* populations were identified, underscoring the value of integrative taxonomic approaches for disentangling the specific biodiversity of this morphologically conservative group of plant-parasitic nematodes. This study reports two newly described species—*L. olearum* sp. nov. and*L. morocciensis* sp. nov.—and the first record of *L. oakgracilis* in Portugal.

All *Longidorus* species are obligate ectoparasites of wild and cultivated plants and are known to cause hypertrophy of root tips. Such damage has been documented in cultivated olives infected by *L. indalus* in southern Spain [28]. However, no apical root galls were observed in any olive trees sampled in this study, regardless of management regime, likely due to the low nematode population densities detected. In contrast, in this study pronounced root tip swelling was observed in globe-fruited retama parasitized by *L. vineacola*.

The present findings parallel those of an earlier large-scale survey across southern Spain (159 locations, 449 samples), in which *Longidorus* was detected in 8.91% of samples and represented by 14 species [28]. Differences in prevalence between the two studies may reflect the smaller sampling effort here, the limited inclusion of natural habitats in which *Longidorus* is more commonly found, and the drier seasonal conditions during this survey. Nonetheless, our results reinforce the need for continued, targeted nematological surveys in olive agroecosystems throughout the Mediterranean Basin.

The results of the present study significantly advance knowledge of *Longidorus* diversity in the region, and suggest that the true species richness of this geneus may be greater than previously reported [3, 4, 11, 28, 49, 62]. The discovery of two novel species and the expanded distribution of *L. oakgracilis* in olives support a dispersalist model to explain *Longidorus* diversification across the Mediterranean Basin. Their comparatively limited spread by human-mediated transport, owing to sensitivity to desiccation, large body size, and the lack of resistant stages, may account for the localized but diverse assemblages observed [3, 8, 11, 28, 49].

Despite continued reliance on morphometrics for taxonomic delimitation, ribosomal and mitochondrial DNA markers have proven essential for accurate identification of *Longidorus* species [3–11, 49]. Our findings reaffirm that the D2–D3 expansion segments of 28 S rRNA provide more robust phylogenetic resolution than does the ITS1 region (due to rapid evolution and poor homology among species) or the highly conserved 18 S rRNA region. While ITS1 is informative for species delimitation, it appears inadequate for resolving phylogenetic structure, likely reflecting high evolutionary rates influenced by various poorly characterized factors.

Phylogenetic analyses based on D2–D3, 18 S rRNA, and COI sequences consistently placed *L. olearum* sp. nov. and *L. morocciensis* sp. nov. within distinct subclades. However, their positions in the COI-based tree were poorly supported and less resolved compared to ribosomal markers. As reported in previous studies, the 18 S rRNA gene displayed high similarity values across *Longidorus* species, limiting its utility in delineating species boundaries or inferring deeper phylogenies [3–11, 49].

Most Mediterranean *Longidorus* species analyzed in this study clustered within Clade I, showing strong agreement with prior phylogenetic frameworks for the genus [3, 4, 28, 52, 63–65]. The newly described species shared moderate body and odontostyle lengths, which may reflect adaptations to feeding on woody hosts such as olive (*Olea europaea*) and globe-fruited retama (*Retama sphaerocarpa*), in line with prior hypotheses linking stylet length to host tissue characteristics [28].

## Conclusion

This study demonstrates the effectiveness of integrative taxonomy for the accurate identification of needle nematodes of the genus *Longidorus* and underscores the rich but underestimated biodiversity of this group in Mediterranean olive ecosystems. The findings expand the distributions of several known species, describe two novel taxa, and provide new sequence data for ribosomal and mitochondrial DNA markers that are critical for species identification. Our work reinforces the importance of further systematic surveys across cultivated and natural habitats in the Mediterranean Basin to uncover the full diversity of this ecologically and economically significant genus. Moreover, the inclusion of juvenile stages and molecular data contributes valuable insights into the biology and ecology of *Longidorus* spp. in agricultural soils.

## Data Availability

All data generated or analysed during this study are included in this published article. Sequences are deposited in GenBank, NCBI. This article has been registered at Zoobank (http://zoobank.org/urn: lsid: zoobank.org: pub:78900B3C-992B-48C8-932B-F2F1AFA5616E).
